# Dystrophic calcification vs sialolithiasis in a pediactric parotid gland: A case report

**DOI:** 10.4317/jced.55621

**Published:** 2019-05-01

**Authors:** Valdir-Meirelles Júnior, Rafael Netto, Maria-Elisa-Rangel Janini, Alexandro B. Azevedo, Vitor-Marcello de Andrade

**Affiliations:** 1 Universidade Federal do Rio de Janeiro - Departamento de Patologia e Diagnóstico Oral - Serviço de Estomatologia - Av. Prof. Rodolpho Paulo Rocco, 325 – 1o andar - Rio de Janeiro - Brazil

## Abstract

Calcinosis is a connective tissue disorder characterized by ectopic calcification in soft tissues. It is subdivided into: dystrophic, metastatic, idiopathic and iatrogenic calcification. The formation of mineralized material in the salivary glands is a common finding in the daily practice of dentists and other specialists. In most cases, this calcification is a sialolith. However, a type of ectopic calcification termed dystrophic calcification is characterized by inappropriate biomineralization in soft tissues and may be associated with trauma, chronic and localized infection or inflammatory diseases. We report a case of a 9-year-old female patient who complained of small nodules in the left parotid region, which begun two years before. Her main complaint was of recurring periods of worsened symptoms characterized by the exacerbation and symptomatic remission of the gland volume with occasional otolaryngologic infections. This study aims to discuss ectopic dystrophic calcification in the parotid gland associated with recurrent infection in children.

** Key words:**Dystrophic calcification, salivary gland, pediatric pathology.

## Introduction

Inappropriate ectopic biomineralization in soft tissues is described as calcinosis and may be divided into dystrophic, metastatic, idiopathic, or iatrogenic calcification (1-6). It is caused by calcium and phosphate deposits in an organic matrix (5,6). When associated with trauma and localized infectious/inflammatory diseases and in the absence of a systemic mineral imbalance, it is described as dystrophic calcification. Calcification can occur in all types of soft tissues, but cardiovascular tissues are particularly susceptible. Metastatic calcifications are the reflection of a systemic imbalance in terms of the metabolism of calcium and other salts, which causes their accumulation in the blood and tissues. They most commonly affect the gastric mucosa, kidneys, lungs, and corneas. Idiopathic calcification occurs in normal tissues with calcium and phosphate serum levels within physiological limits. However, iatrogenic calcinosis is commonly associated with kidney disease and hemodialysis (1-6). Calcifications in soft tissue neoplasms occur in advanced or recurring stages and are formed through metaplasia and may represent a histopathological pattern of these entities (7). Salivary stones, or sialoliths, may be found in any segment of the duct system and the parenchyma of the salivary glands. They are formed by the deposition of calcium salts into a niche composed of organic debris that contains cellular debris, mucin, and/or bacteria (8).

## Case Report

**Figure 1:**
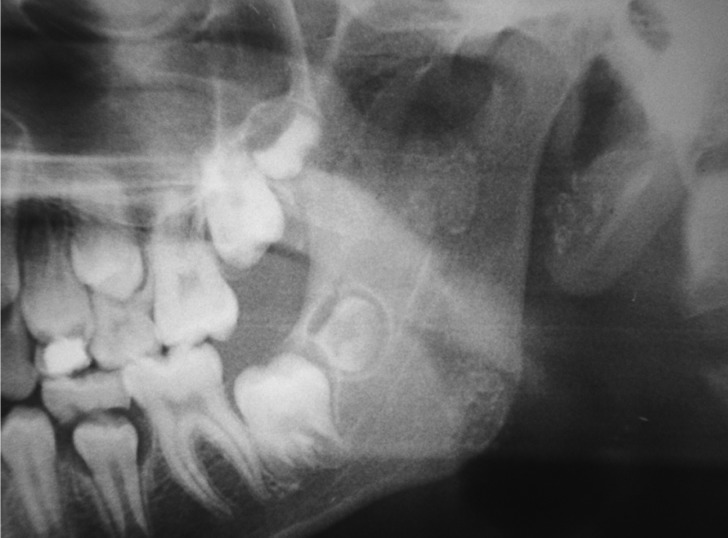
Panoramic Radiography. Notice the multiple radiopaque masses throughout the soft tissue in the parotid region.

**Figure 2:**
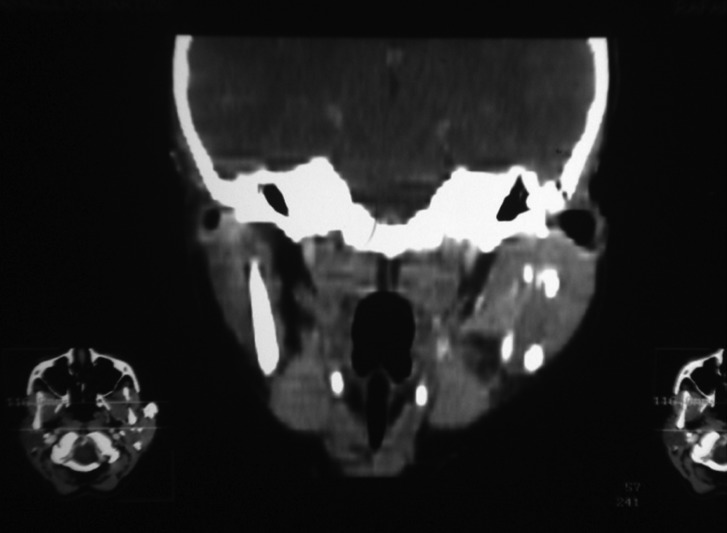
CT Scan – coronal view - dense mass in the left parotid, showing heterogeneous aspect and calcifications in its center.

**Figure 3:**
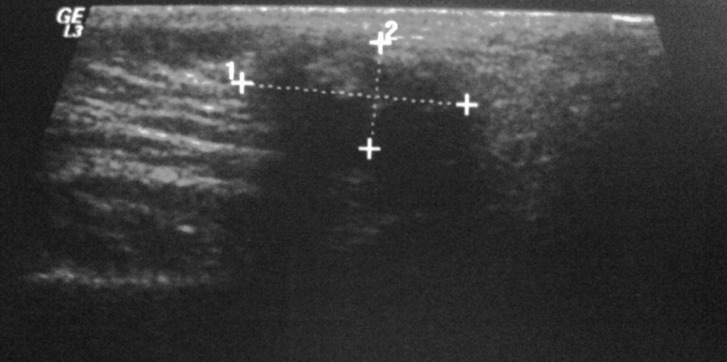
Ultrasound examination showing heterogeneous echotexture of the left parotid but without acute inflammatory foci.

A 9-year-old female leucoderma patient presented to the stomatology department of a public hospital in Rio de Janeiro, Brazil. She complained of small nodules in the left parotid region that had developed over the course of 2 years. Her main complaint was of recurring periods of worsened symptoms characterized by the exacerbation and remission of gland volume that was possibly triggered by occasional otolaryngologic infections or unrelated to these infections. These symptoms suggest juvenile recurrent parotitis. Facial panoramic radiography revealed the presence of multiple circular radiopaque masses in the left parotid region (Fig. 1). The ultrasound revealed increased volume of the left parotid, with imprecise borders, heterogeneous echotexture with hypoechoic and hyperechoic areas within it. These features were suggestive of an inflammatory process associated with calcifications in the parenchyma of the gland. CT scan revealed a dense mass in the left parotid; it was heterogeneous and included calcifications in its center (Fig. 2). Because of the association between the patient’s clinical history, her clinical presentation, and the imaging findings, the possible origin of the calcified materials was questioned. There was evidence of sialoliths or dystrophic calcification associated with recurrent inflammation/infection. Sialoliths are typically symptomatic because of their association with secondary bacterial infections, which are generally treated with systemic antibiotic therapy. Spontaneous remission of bacterial sialadenitis associated with sialoliths is not expected. In addition, sialoliths generally observed as oval-shaped calcified masses or fusiforms on imaging. Because of the pediatric nature of this case, the clinical conduct selected to treat this patient was clinical follow-up and the use of imaging and functional assessments of the gland affected every 6 months or when any signs and/or symptoms appeared. After 48 months, the patient is asymptomatic, without periods of exacerbation of the condition. Recent ultrasound (Fig. 3) demonstrates an improvement in the inflammatory aspect of the gland. Clinical and imaging follow-up will be maintained.

## Discussion

In the literature, there is a wide variety of terminology used to describe the different types of ectopic calcifications in soft tissues, which presents a certain difficulty for academia (1-6). Our approach sought to simplify this abnormal biomineralization process associated with calcium and phosphates for better understanding. Pathological biomineralization in soft tissues is associated with local or systemic factors and may or may not be associated with an imbalance in calcium and phosphate metabolism (9,10). By definition, calcinosis is a disease affecting the connective tissue. It is classified into four types: metastatic, idiopathic, iatrogenic, or dystrophic calcification. Metastatic calcification refers to calcium deposits in normal tissues, with increases in serum calcium and/or phosphate. The idiopathic form of calcinosis affects normal tissues, although serum calcium and/or phosphate levels remain unchanged. Iatrogenic calcification is associated with increases in serum calcium and/or phosphate (1). Dystrophic calcification is characterized by the abnormal deposition of calcium salts in the affected tissue (skin, subcutaneous tissue, glands, muscles, or tendons); serum calcium and phosphate levels remain within physiological limits, though this type is associated inflammatory/infectious processes and localized trauma (1-6). Chronic kidney disease, hypervitaminosis D, prolonged immobilization, and hyperparathyroidism are associated with an increase in serum calcium and phosphates (hypercalcemia) and, thus, with calcinosis (5,6). Metastatic calcification is closely associated with the systemic issues involved in the metabolic imbalance of calcium and phosphate, such as primary hyperparathyroidism and chronic kidney disease (5,6,10). Rarely, cases of hypervitaminosis D, sarcoidosis, melorheostosis, milk-alkali syndrome, and familial tumoral calcinosis may all present this type of ectopic calcification (5). Thus, laboratory testing to determine calcium, phosphate, parathyroid hormone, and vitamin D levels is crucial for the differential diagnoses of the several causes of ectopic calcification that are metabolic in nature; from a histological and immunological perspective, these causes are often indistinguishable (3). In this specific case, there was no evidence of any alterations in mineral metabolism according to the laboratory examinations. Idiopathic calcification affects normal tissues and typically occurs in childhood or adolescence; in this case, serum calcium and phosphate levels remained normal. It may be classified as single or multiple, it may be sporadic, or it may be associated with Down syndrome (11). Iatrogenic calcification may be caused by hypercalcemia associated with hypervitaminosis D, an excess in corticosteroid administration, the intravenous administration of calcium gluconate, and calcium salt deposits on the skin after electromyography or electroencephalography (9). Although rare, ectopic calcifications are most commonly observed around joints and ligaments and in glandular, muscular, or vascular tissue. They are usually associated with local chronic inflammatory processes, scarring, trauma, and autoimmune diseases such as systemic lupus erythematosus (SLE) and dermatomyositis. In this case, the ectopic calcifications were characterized as dystrophic calcifications (1-5). Neoplasms that affect the larger salivary glands, and specifically the parotid glands, may present calcifications. Pleomorphic adenoma is the most common tumor in this area; it may present calcification in advanced or recurring stages, and it generally occurs in adults. In addition, ossifying fibromyxoid tumors in soft tissues, although rare, have been described in the parotid glands and show calcifications (7). However, this neoplasm is rare and primarily affects adults. The case presented herein is different. Dystrophic calcification occurs in the absence of a systemic imbalance in mineral metabolism. It is frequently associated with trauma and recurring infections/inflammations in areas with tissue damage or degeneration. It occurs in any area of soft tissue, although it more commonly affects muscles and cardiac valves. It has been described to affect the masseter and pterygoid muscle and the parotid gland (1,2,4). Cases of recurrent juvenile parotitis cause atrophy of glandular tissue acini and the formation of fibrosis. Collagen is attracted to calcium ions, which initiates its deposition on the protein matrix and may result in dystrophic calcifications in the affected gland (12-14). Recurrent juvenile parotitis may develop because of a parasitic infection, specifically in the case of cysticercosis. It generally occurs in the brain, muscles, or liver as small, half-moon-shaped calcifications (15). Sialolithiasis is caused by excess calcium salts and phosphates around a niche composed of mucus, cellular debris, and/or bacteria in the ducts or parenchyma of the salivary glands, resulting in the formation of salivary calculi or sialoliths. Sialadenopathy is the most common salivary gland disorder in adults; approximately 90% of sialoliths occur in the submandibular gland (8,12-14). Multiple sialoliths in a pediatric patient affecting the parotid gland appeared to be the most probable diagnosis when considered in terms of the patient’s clinical history and the results of the patient’s imaging tests. In this case, the correlation with the recurring and localized tissue damages was evident, and the normal results of the laboratory examinations ruled out autoimmune, metabolic, and parasitic diseases. Thus, dystrophic calcification associated with juvenile recurrent parotitis was diagnosed. Laboratory testing to determine the presence or absence of imbalances in the metabolism of calcium and phosphates in cases of soft tissue calcifications are fundamental for ruling out metastatic calcifications associated with systemic disorders. In addition, tests for autoimmune diseases in cases of soft tissue calcification represent an important tool for this differential diagnosis.
